# Pangenome analyses of the wheat pathogen *Zymoseptoria tritici* reveal the structural basis of a highly plastic eukaryotic genome

**DOI:** 10.1186/s12915-017-0457-4

**Published:** 2018-01-11

**Authors:** Clémence Plissonneau, Fanny E. Hartmann, Daniel Croll

**Affiliations:** 1 Plant Pathology, Institute of Integrative Biology, ETH Zurich, 8092 Zurich, Switzerland; 2 UMR BIOGER, INRA, AgroParisTech, Université Paris-Saclay, Avenue Lucien Bretignières, BP 01, Thiverval-Grignon, F-78850 France; 3 Ecologie Systématique Evolution, Univ. Paris-Sud, AgroParisTech, CNRS, Université Paris-Saclay, 91400 Orsay, France; 4 Laboratory of Evolutionary Genetics, Institute of Biology, University of Neuchâtel, CH-2000 Neuchâtel, Switzerland

**Keywords:** Fungal pathogen, Genome evolution, Genome assembly, Pangenome analyses, Pathogen evolution, *Zymoseptoria tritici*

## Abstract

**Background:**

Structural variation contributes substantially to polymorphism within species. Chromosomal rearrangements that impact genes can lead to functional variation among individuals and influence the expression of phenotypic traits. Genomes of fungal pathogens show substantial chromosomal polymorphism that can drive virulence evolution on host plants. Assessing the adaptive significance of structural variation is challenging, because most studies rely on inferences based on a single reference genome sequence.

**Results:**

We constructed and analyzed the pangenome of *Zymoseptoria tritici*, a major pathogen of wheat that evolved host specialization by chromosomal rearrangements and gene deletions. We used single-molecule real-time sequencing and high-density genetic maps to assemble multiple genomes. We annotated the gene space based on transcriptomics data that covered the infection life cycle of each strain. Based on a total of five telomere-to-telomere genomes, we constructed a pangenome for the species and identified a core set of 9149 genes. However, an additional 6600 genes were exclusive to a subset of the isolates. The substantial accessory genome encoded on average fewer expressed genes but a larger fraction of the candidate effector genes that may interact with the host during infection. We expanded our analyses of the pangenome to a worldwide collection of 123 isolates of the same species. We confirmed that accessory genes were indeed more likely to show deletion polymorphisms and loss-of-function mutations compared to core genes.

**Conclusions:**

The pangenome construction of a highly polymorphic eukaryotic pathogen showed that a single reference genome significantly underestimates the gene space of a species. The substantial accessory genome provides a cradle for adaptive evolution.

**Electronic supplementary material:**

The online version of this article (doi:10.1186/s12915-017-0457-4) contains supplementary material, which is available to authorized users.

## Background

Chromosomal rearrangements facilitate the emergence of evolutionary novelty in eukaryotic genomes [1]. Among other consequences, rearrangements impact adaptive evolution by generating variation in gene content among individuals and through the emergence of new genes. Most gene gains are the result of duplication events, followed by diversification and neofunctionalization [2, 3]. Non-homologous recombination can lead to gains in gene function through the acquisition of new domains. Gene gains have been traced back to horizontal gene transfer events or to de novo gene emergence from non-coding DNA [4, 5]. Gene losses can occur through non-homologous recombination or pseudogenization. Despite being largely under negative selection, gene losses also contribute to adaptive evolution [6]. Gene gains and losses are critical for the rapid adaptation of filamentous plant pathogens to different hosts [7].

In fungi, intra- and interspecific structural variation among genomes has long been recognized [8]. In pathogens, structural variation can have an impact on host range. For example, the host jump from dicotyledon to monocotyledon hosts was accompanied by the loss of genes in the fungus *Melanopsichium pennsylvanicum* [9]. Over longer evolutionary time scales, the expansion or contraction of gene families is tightly associated with host specialization of plant pathogenic fungi [10–12]. In contrast to the ubiquitous evidence for structural variation among genomes of different species, intraspecific analyses of structural variation and polymorphism in gene content are rare outside of a small number of model organisms. Yet, many fungal genomes are amenable to accurate and complete genome assemblies using long-read sequencing technologies [13, 14].

Analyses of multiple complete genomes of the same species have the potential to reveal segregating chromosomal polymorphism and are necessary to accurately cover the gene repertoire of a species. Capturing the full gene repertoire of a species is widely referred to as the pangenome [15, 16]. The pangenome concept distinguishes the core genome (i.e., genes found in all individuals) from the accessory genome (i.e., genes absent in one or more individuals). The distinction between core and dispensable genomic regions is relevant because these compartments are often on distinct evolutionary trajectories [17–19]. Pangenomes constructed over the past decade revolutionized the understanding of genomic variability in bacterial species or lineages. For example, the sequencing of 61 *Escherichia coli* strains showed that only 993 genes are shared among all individuals, while the pangenome is estimated to comprise 15,741 genes [20]. Compared to the relative simplicity of prokaryotic genomes, advances in long read sequencing technologies have been necessary to efficiently assemble eukaryotic genomes [21, 22]. Recently, pangenomes have been constructed for plant species with complex genomes, including maize, *Brassica oleracea*, and soybean species [23–25]. Intra-species genomic analyses of fungi have largely focused on species complexes and on genes missing compared to a reference genome strain [26–29], with the notable exception of Baker’s yeast, where 12 complete genome assemblies are available [30].

Capturing the intra-specific gene content is particularly relevant for plant pathogenic fungi because major determinants of pathogenicity are often encoded by genes not shared among all strains. Plant pathogens and their hosts are often locked in arms races to counter newly evolved host resistance and fungal pathogenicity factors, respectively [31]. To enhance plant colonization, pathogens secrete small effector molecules, which can be recognized by plant resistance proteins to trigger defense mechanisms [32]. In modern agroecosystems, the large-scale deployment of single host genotypes can create uniformity in host resistance mechanisms. Such monocultures exert strong selection pressures on pathogens to evolve effectors that target the deployed host resistance mechanism [33]. Analyses of a broad range of plant pathogens revealed that these rapidly evolving effectors were often located in rapidly evolving compartments of the genome [34]. In filamentous plant pathogens, the compartmentalization of the genome and tight association of effectors and transposable elements in the same compartments has been described as the ‘two-speed genome’ model of pathogen evolution [7]. This bipartite compartmentalization includes blocks of conserved and gene-dense regions and blocks of highly rearranged regions, which are generally gene-poor but enriched in rapidly evolving effector loci. Capturing the pangenome of a pathogen species will enable joint analyses of genome plasticity and yet unknown determinants of pathogenicity.

The plant pathogenic fungus *Zymoseptoria tritici* is responsible for Septoria tritici blotch, one of the most damaging diseases on wheat [35]. *Z. tritici* populations have rapidly evolved resistance to fungicides and have surmounted major wheat resistance genes [36, 37]. Rapid adaptive evolution was likely facilitated by the large effective population sizes, gene flow, and high recombination rates [38–40]. However, the genetic basis of virulence remains poorly understood and, to date, only a few effector genes have been functionally characterized [41–43]. The genome of *Z. tritici* harbors 13 core chromosomes and up to 8 accessory chromosomes, which are not found in all strains of the species. Accessory chromosomes have been shown to undergo major structural rearrangements during meiosis [44, 45]. Homologous core chromosomes show substantial length polymorphism, which is caused by the insertion or deletion of clusters of transposable elements [14, 46].

Gene content can vary considerably among homologous chromosomes. Analyses of two completely assembled genomes showed that hundreds of genes were unique to either of the two genomes [14, 45]. Chromosomal sequences harboring such orphan regions within species (i.e., not shared among all isolates) were also referred to as accessory or dispensable regions [14, 47]. Orphan regions play an important role in adaptive evolution of the pathogen as these regions are enriched in effector genes, including the recently discovered effectors linked to the breakdown of host resistance [42, 48].

We constructed a pangenome of *Z. tritici* by performing de novo complete genome assemblies and analyses of a total of five telomere-to-telomere genomes. Using comprehensive transcriptomics datasets, we annotated the gene space of each newly assembled genome and performed comparative genomics analyses to estimate the total gene content of the species. We extrapolated from the pangenome of five strains to the entire species by analyzing genome-wide polymorphism in a worldwide collection of 123 sequenced isolates.

## Results

### Complete genome assemblies of *Z. tritici* strains

**Fig. 1:**
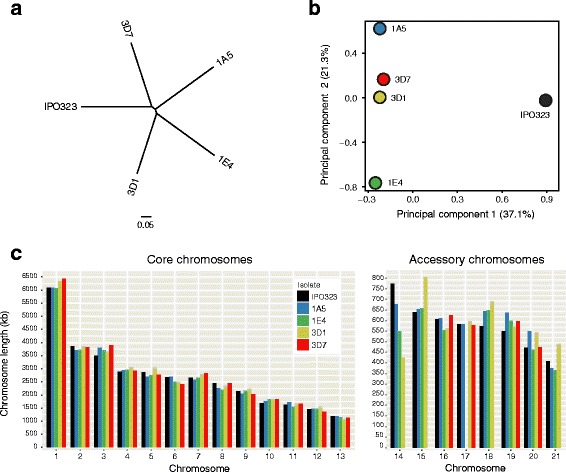
Comparison of complete genome assemblies of five *Zymoseptoria tritici* strains used to construct the pangenome. **a** Maximum likelihood phylogeny of the isolates based on a genome-wide single nucleotide polymorphism (SNP) matrix. The scale refers to the proportion of total SNPs included in the analysis. **b** Principal component analysis based on a genome-wide SNP matrix. The variance explained by each of the two axes is shown in parentheses. **c** Lengths of completely assembled core and accessory chromosomes are shown in separate panels

**Fig. 2:**
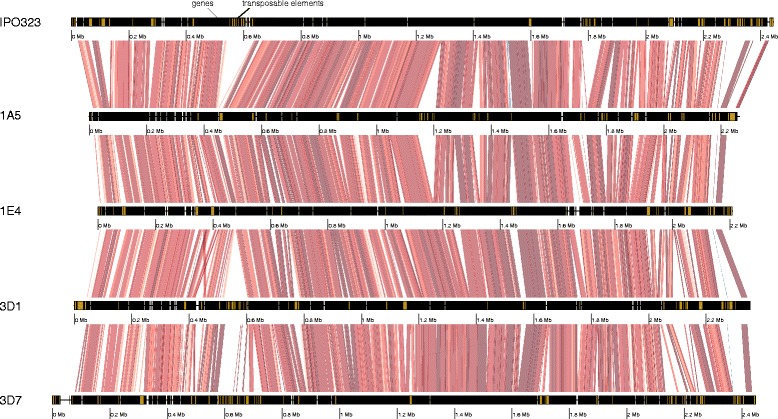
Pairwise chromosomal synteny between homologs of chromosome 8. Collinear sequences are shown by red segments with light to dark red indicating levels of sequence identity (90–100%). Light to dark blue indicates levels of sequence identity (90–100%) of inverted sequences. The locations of genes and transposable elements are shown in black and yellow, respectively. Chromosomal positions are shown with separate megabase (Mb) scales

We used high-coverage PacBio sequencing to assemble complete chromosomes of the *Z. tritici* strains 1A5, 3D1, and 3D7. These three strains and the two strains for which complete genomes were already available had approximately identical phylogenetic distances (Fig. 1a). A principal component analysis revealed that strains 1A5, 1E4, 3D1, and 3D7 were genetically more similar to each other than the reference genome isolate IPO323 (Fig. 1b) reflecting the geographic origin of the different strains. High-density genetic maps available for the same isolates [49] confirmed the contiguity of all assembled chromosomes. Illumina read mapping to each genome assembly revealed ≤ 12 single base or small indel errors, which were corrected according to the evidence from the Illumina read data. The three new genomes comprised 20 (1E4) or 21 (1A5 and 3D1) chromosomes (Fig. 1c). The two previously available genomes comprised 17 (3D7) and 21 chromosomes (reference genome IPO323). The total size of the five genomes ranged from 37.9 to 40.7 Mb (Additional file 1: Table S1). Differences in genome sizes were due to a combination of differences in accessory chromosome numbers (4–8) and chromosome length polymorphism (Fig. 1c). Core chromosome length polymorphism ranged from 2.1% to 4.8%, whereas length polymorphism among accessory chromosomes reached up to 25% (Additional file 1: Table S1). Length variation among homologous chromosomes was caused by substantial insertion and deletion polymorphism in both genic and non-genic regions (Fig. 2).

### Gene prediction and comparative analyses of gene content

Comparative genomics analyses of the two previously completed genomes 3D7 and IPO323 conservatively estimated that each genome harbored 296 and 216 gene sequences, respectively, that had no homology in the other genome [14]. To identify protein coding genes in the assembled genomes, we performed gene prediction analyses using splicing evidence from transcriptomic datasets collected from each of the isolates over the course of a complete wheat infection cycle. To ensure that functional genes were consistently identified across all genomes, we used matches of IPO323 proteins against all other genomes as additional evidence during gene prediction.

The total number of encoded proteins identified in each of the five genomes ranged from 11,737 to 12,092 (Table 1). We evaluated the completeness of both the genome assemblies and the quality of the gene predictions by performing BUSCO analyses on the predicted proteins. We found that all genome annotations were highly complete, ranging from 96.8% to 98.3% (Additional file 2: Table S2). We identified a similar number of conserved proteins in each isolate (7353 to 7439). The number of proteins predicted to be secreted (secretome) ranged from 932 to 1059 among isolates (Table 1, Additional file 3: Table S3). We analyzed the set of proteins likely acting as effectors in host interactions based on predictions from EffectorP [50]. We found a total of 221 candidate effectors in the genome of IPO323 and a range of 286–330 candidate effectors among the four Swiss isolates 1A5, 1E4, 3D1, and 3D7 (Table 1).

**Table 1:** Genome assembly and annotation statistics of the five complete *Zymoseptoria tritici* genomes

Genomes assembly	1A5	1E4	3D1	3D7	IPO323
Genome size, Mb	39.7	38.6	40.7	37.9	39.7
Chromosomes, n	21	20	21	17	21
Gene annotation					
Predicted genes	12,092	12,033	12,006	11,737	11,839
Average gene length, bp	1520.2	1524.2	1514.1	1502.6	1620.9
Average protein length, aa	467.5	468.3	466.4	457.2	487.8
Number of exons	29,716	30,015	29,033	30,399	30,068
Average exons per gene	2.46	2.49	2.42	2.59	2.54
Average exon length, bp	570.7	563.3	579.8	530.7	575.2
Number of introns	17,628	17,984	17,027	18,663	18,226
Average introns per gene	2.18	2.21	2.15	2.29	2.54
Average intron length, bp	80.7	79.7	79	80.6	91.6
Genes with introns	8099	8129	7925	8142	8044
Gene density, Mb^–1^	304.6	311.7	294.9	309.7	298.3
Protein functions					
Proteins with Pfam domain	7403	7439	7431	7353	7375
Secreted protein	1025	1059	1014	1034	932
Predicted effectors^a^	309	286	296	330	221

^a^Effectors were predicted using the software EffectorP [50]

### Pangenome analyses of core and accessory gene sets

**Fig. 3:**
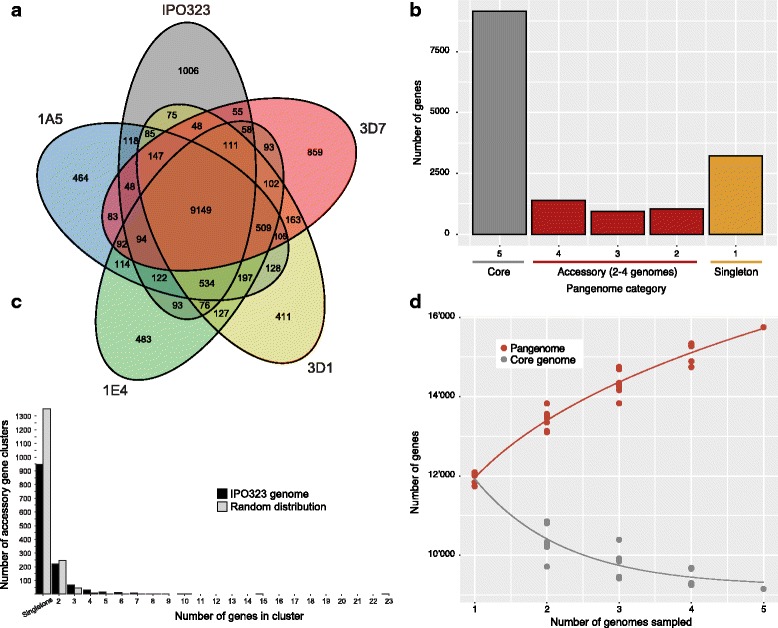
The pangenome of *Zymoseptoria tritici*. **a** Venn diagram of the singleton, accessory, and core genes of the pangenome. **b** Categorization of the pangenome into singleton, accessory, and core genes according to the number of times a specific gene was identified among genomes. **c** Genes of the isolate IPO323 classified as either accessory or singleton in the pangenome were analyzed for clustering in the genome (black bar). In comparison, the mean of 1000 randomly drawn distributions of accessory genes are shown with grey bars. Accessory genes were significantly more likely to be in clusters than expected by chance (Fisher exact test, *P* < 0.0001). **d** Estimation of the sizes of the core genome (genes shared among all isolates) and the pangenome (all genes). Genomes were resampled in all possible combinations of 1–5 and the number of core and pangenome genes are reported as dots. The pangenome curve was modelled by fitting the power law regression formula y = Ax^B^ + C and the core genome curve was modelled by fitting the exponential regression formula y = Ae^Bx^ + C

In order to assess shared protein functions among isolates, we constructed a pangenome by clustering the protein sets encoded by each of the genomes of isolates 1A5, 1E4, 3D1, 3D7, and IPO323. The thresholds used at the protein clustering stage affected the number of detected homologs. We conservatively considered proteins with more than 60% length and more than 75% sequence similarity as homologs. Decreasing the thresholds did not meaningfully reduce the size of the pangenome, whereas increasing the thresholds increased the pangenome size by approximately 20% (Additional file 3: Table S3). We identified a total of 15,749 non-redundant proteins, of which 9149 (58.1%) were shared among all five analyzed genomes (Fig. 3a, Additional file 4: Table S4). We defined this set of proteins to be encoded by the core genome. The accessory genome of all strains combined encoded 6600 (41.9%) non-redundant proteins. The accessory genome encoded 3377 (21.4%) proteins shared by at least two isolates and 3223 (20.4%) singleton proteins found in only one isolate (Fig. 3b). IPO323 and 3D7 had a higher proportion of singleton proteins, with 1006 and 859 proteins, respectively (Fig. 3a). Isolates 1A5, 1E4, and 3D1 had comparable numbers of singleton proteins (411–483). The number of singleton proteins reflected the genetic distances among the isolates as shown by the principal component analysis (Fig. 1b).

Among the 15,749 proteins encoded by the pangenome of *Z. tritici*, only 76 proteins had a paralog. This low number of paralogs may be a consequence of efficient repeat-induced point (RIP) mutations introduced into any recently duplicated sequence [51]. Most paralogs originated from simple duplication events (Additional file 5: Table S5). Three of the paralogs (Cluster_002657, Cluster_007468, and Cluster_009805) encoded candidate effectors. The most expanded paralog families were Cluster_001114, Cluster_000001, Cluster_000201, Cluster_000202, and Cluster_000200, and most paralog members were found in the 1E4 and 1A5 genomes with up to 13 and 24 copies, respectively (Additional file 5: Table S5).

Differences in encoded proteins among isolates can stem from several factors, including gene gains and losses, as well as segregating loss-of-function (LOF) mutations. We evaluated IPO323 proteins for which we found no pangenome homolog in either one of the four other isolates. Thus, such genes were classified as accessory or singleton proteins. We mapped Illumina whole genome sequencing data from each of the four other isolates to the genome of IPO323 and analyzed sequence coverage. We found that 31–35% of the genes for which an isolate was not predicted to have a functional protein lacked sequence coverage (Additional file 6: Table S6, Additional file 7: Figure S1). We found that an additional 46–51% had at least partial sequence coverage but contained LOF mutation causing protein truncations (Additional file 6: Table S6). Finally, we mapped RNAseq data of each isolate to the genome of IPO323 and quantified expression levels of the accessory genes. We found that, overall, 80–83% of all genes lacked transcription over the course of an infection (Additional file 6: Table S6). In summary, isolates lacked accessory proteins due to a combination of gene deletions and LOF mutations. Additional factors may be related to pseudogenization, as suggested by a lack of transcription. In summary, these factors accounted for a total of approximately 90% of accessory genes in the pangenome (Additional file 6: Table S6).

### Structure and function of the pangenome

**Fig. 4:**
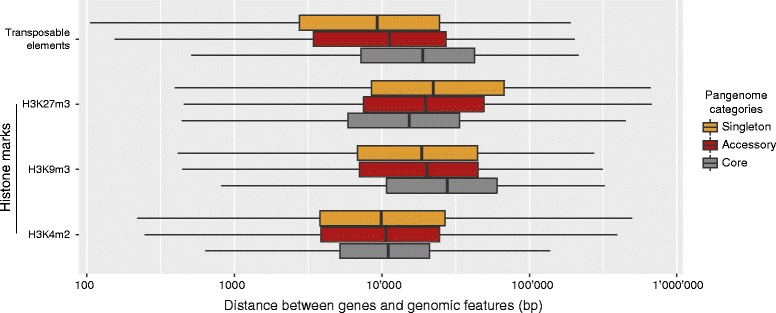
Association of core, accessory, and singleton genes in the pangenome with transposable elements and chromatin states. The genome of IPO323 was analyzed for specific histone methylations associated with euchromatin (H3K4me2), obligate (H3K9me3), and facultative (H3K27me3) heterochromatin regions. The boxplots show the median physical distances in bp

We found that accessory and singleton genes were frequently clustered into blocks of 2–23 genes in the IPO323 genome (Fig. 3c). The degree of clustering was significantly higher than expected from a random distribution of gene localizations (Fisher exact test, *P* < 0.0001). In the IPO323 genome, singleton genes were closest to transposable elements followed by accessory and core genes (Fig. 4). As an evolutionary response to prevent transposable element proliferation, regions rich in transposable elements can be silenced through histone lysine methylation. Specific methlyations are associated with euchromatin (H3K4me2), obligate (H3K9me3), and facultative (H3K27me3) heterochromatin regions. All pangenome gene categories were at similar distances to euchromatin regions (Fig. 4). Mirroring the association with transposable elements, singleton genes were closer to regions of obligate heterochromatin. In contrast, core genes were closer to regions of facultative heterochromatin (Fig. 4). In the *Z. tritici* genome, facultative heterochromatin was mostly associated with subtelomeric regions, while obligate heterochromatin was enriched in regions rich in transposable elements.

We estimated the total size of the core and pangenome by resampling 1–5 genomes and reporting the size of the core and pangenome. We found that the core genome likely stabilizes at approximately 9000 core genes (Fig. 3d). However, the number of accessory genes discovered with each additional genome did not stabilize and the pangenome size increased almost linearly with the number of genomes (Fig. 3d). Thus, the complete pangenome of the species is likely substantially larger than estimated from these five genomes.

**Fig. 5:**
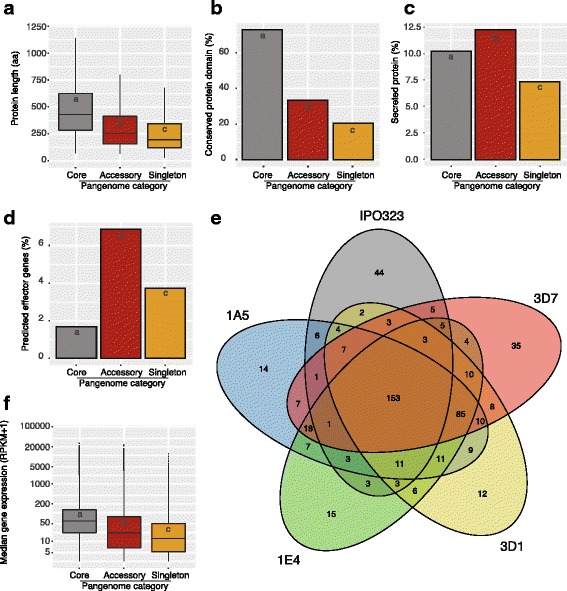
Functional analyses of the *Zymoseptoria tritici* pangenome*.* **a** Lengths of the encoded proteins by core, accessory, and singleton genes of the pangenome (different letters are *P* < 0.0001, Kruskal–Wallis test). **b** Percentage of proteins with a conserved protein domain (different letters are *P* < 0.0001, Fisher exact test). **c**, **d** Percentage of genes encoding secreted proteins and candidate effectors, respectively (different letters are *P* < 0.0001, Fisher exact test). **e** Venn diagram of shared and unique effector candidates in the pangenome of isolates 1A5, 1E4, 3D1, 3D7, and IPO323 (reference genome). **f** Median expression levels of pangenome genes (different letters are *P* < 0.0001, Kruskal–Wallis test)

We analyzed three categories of genes constituting the pangenome of *Z. tritici*, namely core genes shared among all five genomes, accessory genes found in 2–4 genomes, and singleton genes found only in a single genome. We defined these categories to approximate the gene frequencies in the species and identify specific characteristics of subsets of the pangenome. The average amino acid sequence length was significantly different between the three categories, with core genes encoding on average the longest amino acid sequences (Kruskal–Wallis test, *P* < 0.0001, Fig. 5a). Among core genes, 67% encoded a conserved protein domain. The percentage decreased to 32% and 20% for accessory and singleton genes (Fig. 5b). Pathogen genomes encode a large number of secreted proteins with roles in host interactions. We found that both secreted proteins and effector candidates were significantly enriched among proteins encoded by accessory and singleton genes, with the highest proportion found in accessory genes (Fig. 5c, d). We identified a total of 153 core effector candidates shared among all five genomes as well as 232 accessory and 120 singleton effector candidates (Fig. 5e). The genomes of IPO323 and 3D7 encoded the largest number of singletons with 44 and 35 effector candidates, respectively. Levels of gene transcription also varied among gene categories with core genes showing the highest and singletons showing the lowest median transcription levels during infection of wheat (Fig. 5f).

We analyzed whether different gene categories of the pangenome encoded distinct protein functions. For this, we performed gene ontology (GO) term enrichment analyses. We found that core genes were significantly enriched in housekeeping genes including basic cellular functions and development. Core genes were also significantly enriched in genes involved in general metabolism functions such as lipid, sugar or amino acid metabolism, biosynthesis and cell cycle (Additional file 8: Figure S2, Additional file 9: Figure S3 and Additional file 10: Figure S4). We found distinct GO terms to be enriched in accessory and singleton gene categories. Accessory genes were significantly enriched in functions such as transmembrane transporter activity (Additional file 10: Figure S4). Moreover, we found enrichment in cellular membrane localization (Additional file 8: Figure S2). We also identified an enrichment in zinc ion binding functions and protein kinase activity, which suggests that the encoded proteins play a role in signaling and transcriptional regulation. Singleton genes were most significantly enriched in functions related to DNA integration processes (Additional file 8: Figure S2). Such functions often originate from the insertion of transposable elements in protein coding genes. We also found significantly enriched functions related to host adaptation, including defense responses, chitin catabolic processes, as well as hydrolase and cysteine-type peptidase activities.

### Pangenome characteristics in a worldwide collection of *Z. tritici*

**Fig. 6:**
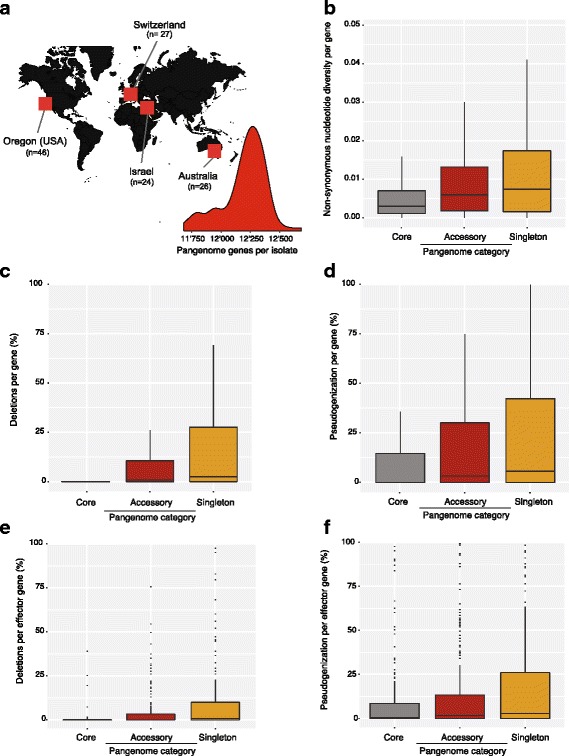
Population genomic analyses of different pangenome gene categories. **a** Illumina whole-genome sequencing data of 123 *Zymoseptoria tritici* isolates covering the worldwide distribution range was analyzed for polymorphism in core, accessory, and singleton genes of the pangenome. The distribution shows the number of pangenome genes detected in each isolate. Gene presence was defined as showing no evidence for deletion (based on sequence coverage) and no loss-of-function (LOF) mutations (based on variant calling). **b** Non-synonymous nucleotide diversity (π_n_) per gene. **c** Percentage of isolates showing evidence for a gene deletion. **d** Percentage of isolates showing evidence for LOF mutations. **e** Percentage of isolates showing evidence for an effector gene deletion. **f** Percentage of isolates showing evidence for LOF mutations in effector genes

We investigated whether the distinct properties of the pangenome gene categories were representative of species-wide patterns in gene presence/absence variation and LOF mutations. For this, we analyzed polymorphism detected in a worldwide collection of 123 *Z. tritici* isolates. We mapped Illumina short-read sequencing data of all isolates to each of the five completely assembled genomes (Additional file 11: Table S7). Taking the 15,749 non-redundant genes of the pangenome as a reference, we determined, for all isolates, whether the pangenome genes were present and functional (lacking frameshift mutations). We found that each of the 123 isolates encoded between 11,683 and 12,684 functional genes (Fig. 6a). Non-synonymous nucleotide diversity was significantly lower in core genes than in accessory genes (Kruskal–Wallis test *P* < 0.0001; Fig. 6b, Additional file 12: Table S8). Next, we analyzed copy number variation based on mapped read depth using CNVnator. Among all 123 isolates and all 15,749 non-redundant genes of the pangenome, we detected a total of 113,350 individual gene deletions. A total of 4556 loci showed evidence for a complete gene deletion in at least one isolate. We found strong differences in the rate of gene deletions between the core and accessory genome (Fig. 6c). Only 7.8% of the core genome loci showed evidence for deletions among the global collection of isolates, but 55.3% and 61.2% of the accessory and singleton genome loci, respectively, showed evidence for deletions. The majority (52%) of the gene deletion loci had a low deletion frequency among isolates (<10%). Core genome loci had the lowest deletion frequencies with an average of 1.2% (Fig. 5a). Accessory and singleton genes showed higher frequencies of individual gene deletions, with averaged frequencies of 9.6% and 16.8%, respectively.

Pseudogenization often precedes gene loss and could generate additional variation in gene content within the species. We investigated two dominant causes of pseudogenization, namely premature stop codons and frameshift mutations. To identify these two types of LOF mutations, we analyzed single nucleotide polymorphisms (SNPs) and small indels segregating among the 123 isolates. As for the coverage analyses above, we called polymorphisms independently using reads mapped against each of the five completely assembled genomes (Additional file 6: Table S6). We found LOF mutations in a total of 8669 genes spanning all gene categories of the pangenome. Overall, 45.4% of the core genes, 68.6% of the accessory genes, and 68.2% of the singleton genes had at least one LOF mutation in at least one isolate. The frequency of LOF was significantly higher in accessory genes compared to core genes (Kruskal–Wallis test, *P* < 0.0001; Fig. 6d).

### Pangenome analyses of effector genes

In *Z. tritici*, two major effector genes were identified using association mapping [42, 43]. The first effector *Zt_8_609* caused avirulence on the wheat cultivar Toronit and was found to segregate a presence-absence polymorphism within the species. Consistent with previous analyses showing that *Zt_8_609* was missing in 1A5, 1E4, 3D1, and 3D7 [42], the gene was identified as a singleton in IPO323 (Additional file 3: Table S3). The effector *AvrStb6* causing avirulence on cultivars carrying the resistance gene *Stb6* was known to be missing in the annotation of IPO323 [43]. However, the gene was recovered and clustered at the protein level in the annotations of 1A5, 1E4, 3D1, and 3D7 (Additional file 3: Table S3).

We more broadly analyzed gene deletions and LOF mutations affecting genes that encoded candidate effectors. The pangenome analyses identified a total of 591 candidate effectors, of which 153 belonged to the set of core genes (Fig. 5e). We analyzed the polymorphism of candidate effector genes in the collection of 123 worldwide isolates. We observed a similar pattern for effector genes than for all gene categories combined. Effector genes showed much stronger conservation within the species if they were core genes. Accessory and singleton effector genes had both higher rates of gene deletions and LOF mutations (Fig. 6e, f). We identified a total of 134 effector genes without any evidence for gene deletion or LOF mutations in any of worldwide isolates. Among these highly conserved effectors, 8 were also conserved at the amino acid level and showed strong up-regulation during plant colonization. This small proportion of highly conserved effectors likely play an essential role in pathogenicity.

## Discussion

We constructed a pangenome for the highly polymorphic wheat pathogen *Z. tritici* based on five completely assembled and independently annotated isolates. We identified a much larger gene repertoire than previously known from the reference genome alone. The core and the accessory genome showed distinct functional, structural and regulatory characteristics. We confirmed the distinct properties of the core and accessory genome by analyzing sequence data of a worldwide collection of 123 additional isolates. Accessory genes were more likely to be polymorphic, contain frameshift mutations and more likely to be deleted compared to core genes. We identified eight highly conserved effector candidates within the species that may play an essential role in pathogenesis.

### A pangenome to approximate the complete gene repertoire of the species

Many fungal species vary considerably in genome size among closely related species and show chromosome length polymorphism within species [34, 52]. Early evidence for chromosome length polymorphism in *Z. tritici* was found based on gel electrophoresis [46]. A recent population genomics study showed that *Z. tritici* populations harbor extensive gene deletion polymorphisms affecting 15% of the genes identified in the reference genome [53]. A comparison of two completely assembled genomes showed that either strain harbored a similar number of additional genes compared to the other strain [14]. Our pangenome construction confirmed that *Z. tritici* harbors a significantly larger gene repertoire than known from the reference genome alone. The gene space of the reference genome IPO323 was estimated to be composed of 11,795 complete genes [54] and our minimum estimate of the gene repertoire of the pangenome was 15,749 genes.

The total number of distinct genes encoded in *Z. tritici* genomes is likely to be substantially higher for two reasons. First, our estimates were based on four Swiss and one Dutch isolates, which are unlikely to fully represent the global polymorphism of the species. Nevertheless, single field populations of *Z. tritici* harbor a substantial portion (~90%) of the total genetic variation within the species due to high effective population sizes and gene flow [38]. Second, our resampling estimates of the total gene content did not stabilize at a plateau with five genomes.

The open nature of the *Z. tritici* pangenome means that a substantial number of additional genes will be identified by assembling additional genomes. In particular, analyzing genomes from the genetically diverse populations in the Middle East and Fertile Crescent, where the pathogen was co-domesticated with wheat, is likely to drastically increase the size of the pangenome. In contrast to the open pangenome, we obtained a confident estimation of the core genome size. Our resampling showed that including additional genomes is unlikely to substantially reduce the set of genes shared within the species. We confirmed this prediction by analyzing the gene content of 123 worldwide isolates and indeed found that more than 98% of all genes defined as core genes were present in all isolates. Core genes also shared a number of properties indicative of their essential role for the organism, and were significantly enriched among others in functions related to basic cellular processes and growth. Loss of genes encoding essential functions would be strongly deleterious.

In contrast to the core genome, the accessory genome was enriched in genes playing important roles in pathogenicity. Plant pathogen genomes encode effectors that can manipulate host immune defenses and largely determine the outcome of infections. Thus, comprehensive knowledge of effector gene loci is crucial to gain insights into the infection process. We identified hundreds of effector genes in the newly assembled genomes of isolates 1A5, 1E4, and 3D1, which were not present in the previously assembled genomes of IPO323 and 3D7 [14, 45, 54]. We identified 250 effector genes that were lacking in the IPO323 reference genome, which is greater than the total number of effectors encoded by the IPO323 genome (221). However, differences in effector gene predictions may also be influenced by the annotation strategy [55]. Predictions for IPO323 were performed using evidence for gene models extracted from transcriptomic data of *Z. tritici* growing on culture medium and during the earliest infection stages [54]. The newly assembled genomes were annotated using transcriptomic evidence across the entire infection cycle [56]. This difference may indeed be important because the expression of many effector genes coincides with the development of large scale lesions and the onset of pathogen reproduction [56]. An example of a missed major avirulence effector is *AvrStb6* [43]. The effector gene was missing from genome annotations of the IPO323 reference genome until evidence for the gene was identified using more comprehensive transcriptomic data. In contrast, the second known avirulence effector, *Zt_8_609*, was properly annotated in the reference genome IPO323, but the gene is deleted in all newly assembled genomes [42]. Our pangenome analyses significantly expanded the effector gene repertoire of *Z. tritici* and provides an essential resource to identify yet unknown avirulence factors.

### Birth and death of genes in the pangenome

The pangenome constructed for *Z. tritici* comprised 42% accessory genes (i.e., not fixed within the species). A more expansive analysis of the pangenome covering more genomes would increase this proportion even further. The extent of the accessory genome raises significant questions with regard to the evolutionary origin of this polymorphism, its role in adaptive evolution, and the trajectory of potentially redundant gene functions. A substantial proportion of the *Z. tritici* accessory genome was generated through LOF mutations segregating among isolates. These mutations were predominantly deleterious frameshifts and early stop codons causing protein truncation. Even if expressed, truncated proteins are unlikely to perform an identical biological function compared to the full-length protein variant and, thus, contribute to functional variation among isolates. Presence-absence polymorphism of genes accounted for slightly less accessory genome polymorphism than LOF mutations. Our analyses of a worldwide collection of isolates confirmed the major characteristics of the pangenome. In addition to higher frequencies of gene deletions, accessory genes were more likely to segregate LOF mutations. We also found that accessory genes had higher non-synonymous nucleotide diversity, which is indicative of relaxed selection caused by functional redundancy.

In a previous analysis of *Z. tritici*, 1623 segregating gene presence-absence polymorphisms were investigated for their evolutionary origins [53]. Interestingly, approximately two-thirds of these polymorphisms were generated by deletions of genes that were shared among closely related species. One-third of the presence-absence polymorphisms affected genes lacking homologs. Therefore, these genes were likely recently gained and had not yet reached fixation within the species. In principle, loss of essential gene functions should be under strong negative selection in haploid organisms [57]. Given the size of the *Z. tritici* accessory genome, a substantial fraction of all *Z. tritici* genes are likely functionally redundant or have no specific function (i.e., the genes are dispensable).

Functional redundancy is unlikely to have evolved by individual gene duplications or whole genome duplications. In fact, both our analysis of the pangenome and previous analyses of the reference genome IPO323 revealed little evidence for paralogy [45]. A likely explanation for the lack of paralogs is that the genome of *Z. tritici* encodes the machinery for the genomic defense mechanism RIP. RIP introduces random mutations in any near identical sequence copy in the genome [58]. RIP primarily evolved as a defense against the activity of transposable elements, but it also constrains evolution by gene duplication [51]. Thus, if gene dispensability evolved as a consequence of redundancy, genes performing similar functions are unlikely to be related by common descent. Functional redundancy in absence of paralogy could explain why many effector gene knockouts have no detectable impact on pathogenicity [59, 60]. Importantly, functional redundancy in pathogen effectors can serve the evolutionary purpose of a ‘bed-hedging’ strategy. A change in the environment, such as the introduction of a host detecting a specific effector, will impose strong selection on the pathogen population to eliminate this effector. Thus, a pathogen population that is highly heterogeneous in effector complements can recover from such selection by favoring non-recognized effectors that converge on the same function as a recognized effector [61].

In addition to redundancy, environmental specificity or local adaptation can be a driver of gene dispensability and, ultimately, gene loss [62]. Some accessory genes could play a role under specific environmental conditions and, thus, contribute to local adaptation. As for functional redundancy, the strongest candidate genes for such environmental specificity would be effector genes that evolved to promote infection on specific host genotypes. Wheat cultivars are genetically highly diverse and encode at least 21 distinct resistance loci against the pathogen [63]. It is thought that most wheat resistance loci are matched by a specific pathogen effector. A pathogen expressing the matching effector gene is unable to colonize the host. Thus, geographic heterogeneity in the deployment of wheat genotypes would favor specific effector gene losses in specific regions. Evidence for such an adaptive gene loss was found for a *Z. tritici* effector gene [42].

## Conclusions

*Z. tritici* populations maintained very significant levels of standing genetic variation as indicated by the speed of adaptive evolution and decay in linkage disequilibrium [37, 39, 40]. Effective population size is an important determinant of pangenome sizes [64]. In absence of selection, large populations should maintain pangenomes by preventing accessory genes to be lost from the gene pool by random drift. A constraint in the expansion of the pangenome likely entails the necessity for homologous chromosome pairing during meiosis. As the *Z. tritici* accessory genome was associated with substantial structural variations, some accessory regions may already show reduced recombination rates. In sufficiently large populations, at least a fraction of the accessory genes could have recently evolved from non-coding DNA. Indeed, de novo gene evolution is common in the model fungus *Saccharomyces cerevisiae* and other eukaryotes [5, 65]. At an early stage, de novo genes would likely have no function and show weak expression, characteristics shared by many genes of the accessory genome. Retracing the evolutionary origin of accessory genes will provide significant insights into the emergence of highly polymorphic pangenomes and their role in adaptive evolution [23, 27, 28, 66, 67]. The analyses of eukaryotic pangenomes will elucidate how major evolutionary novelty arises within species.

## Methods

### High molecular-weight DNA extraction and single molecule real-time (SMRT) sequencing

The isolates ST99CH_1A5, ST99CH_1E4, and ST99CH_3D1 (abbreviated here as 1A5, 1E4, and 3D1, respectively) were sampled from two Swiss wheat fields in 1999 [68]. In order to extract high molecular-weight DNA, we used the modified version of the cetyltrimethylammonium bromide DNA extraction protocol described in Plissonneau et al. [14]. In brief, fungal spores were harvested after 5–6 days in liquid yeast sucrose broth and lyophilized. Approximately 60–100 mg of lyophilized spores were crushed with a mortar and pestle. From a phenol-chloroform-isoamyl alcohol solution, the supernatant was transferred and centrifuged. The pellet was resuspended in phenol-chloroform-isoamyl alcohol and repeated twice. For each isolate, PacBio SMRTbell libraries were prepared using > 15 μg of high molecular-weight DNA. Sequencing was performed using P6/C4 chemistry on a PacBio RSII instrument at the Functional Genomics Center, Zurich, Switzerland.

### Complete genome assemblies

We used the pipeline developed previously for the *Z. tritici* isolate 3D7 [14]. In summary, raw sequencing reads were assembled using HGAP v 3.0 included in the SMRTanalysis suite (version 2.3.0, patch 3) [21]. All settings were at default, with the exception of the minimum seed read length to initiate the self-correction. Different seed read length cut-offs were tested to compensate for pre-assembly yield. The assembled contigs were polished using Quiver with default settings as implemented in the SMRTanalysis suite. Problematic contigs were identified by the mapped PacBio read coverage. Contigs were discarded if the mean mapped coverage deviated by more than 1.5× from the median coverage of all contigs (weighted by length). We previously generated and genotyped crosses of 1A5 × 1E4 and 3D1 × 3D7, respectively [49]. Thus, we were able to evaluate the contiguity of contigs using the high-density SNP-based genetic maps. All assembled contigs were uniquely assigned to a single linkage group. The genetic and physical marker orders were highly correlated. Completely assembled chromosomal sequences were quality checked using short read data available for each of the strains (NCBI Short Read Archive accessions SRS383142, SRS383143, and SRS383146). We used the PILON procedure to map Illumina short reads and corrected indels and SNPs detected in the Illumina read alignments [69]. See Plissonneau et al. [14] for more details on the SNP calling, map construction, and assembly validation procedure.

### Gene prediction and genome annotation

To accurately predict the gene space of each newly assembled genome, we used the gene prediction software BRAKER v1.9 [70]. BRAKER combines coding sequence and intron hints based on the mapping of conserved protein sequences and introns identified in RNA-seq data, respectively. We used RNA-seq datasets generated for isolates 1A5, 1E4, and 3D1 [56], which covered four time points (7, 12, 14, and 28 days after inoculation) over the course of an infection of the susceptible wheat cultivar Drifter. All sequencing datasets were retrieved from the NCBI Short Read Archive under the project accession number SRP077418. Raw RNA-seq reads were quality filtered using Trimmomatic v 0.36 [71] using the following parameters: ILLUMINACLIP:TruSeq3-PE.fa: 2:30:10 LEADING:10 TRAILING:10 SLIDINGWINDOW:5:10 MINLEN: 50, and then aligned to the corresponding genome assembly using tophat v 2.0.14 [72] with the following parameters: --min-intron-length 10 --max-intron-length 1000 --mate-inner-dist 180 --mate-std-dev 40. To extract predicted intron splice sites, we used the tool bam2hints (--minintronlen = 10 --maxintronlen = 1000 --maxgaplen = 9) from the AUGUSTUS v3.2.1 gene prediction software [73]. In addition to intron hints, we used coding sequences annotated in the IPO323 reference genome [54]. For this, we mapped the predicted protein sequences to each assembled genome using exonerate 2.2.0 [74], with the following parameters: --percent 95 --minintronlen = 10 --maxintronlen = 1000 --model = protein2genome. Finally, intron on coding sequence hints were combined and provided to BRAKER to generate gene predictions for each assembled genome.

### Gene expression analyses

We quantified gene expression profiles over the course of a wheat infection using RNAseq datasets generated by Palma-Guerrero et al. [56] for isolates 1A5, 1E4, 3D1, and 3D7, and by Rudd et al. [59] for the reference genome isolate IPO323. RNAseq reads were mapped to the corresponding reference genome using tophat v 2.0.14 [72] using the following parameters: --min-intron-length 10 --max-intron-length 1000 --mate-inner-dist 180 --mate-std-dev 40. To obtain read counts for each gene, we used HTSeq v0.7.1 [75] with the parameters -s reverse –m union. We calculated normalized counts per million and reads per kilobase of transcript per million mapped reads using the R package edgeR normalizing read counts individually per isolate across all time points [76].

### Annotation of gene functions

The completeness of the assembled genomes and the corresponding gene annotations was tested using the Ascomycota dataset of BUSCO version 3 [77]. Predicted proteins were assigned to protein families (Pfam) and GO terms using InterProScan 5.18–57 [78]. Secretion signals, as well as cytoplasmic, transmembrane, and extracellular domains, were predicted using a combination of SignalP 4.1 [79], Phobius 1.01 [80], and TMHMM 2.0 [81]. To define the secretome of each isolate, we selected proteins, which were assigned a secretion signal by both SignalP and Phobius. Proteins with predicted transmembrane domains (by Phobius and TMHMM), an extracellular domain or a cytoplasmic domain (by Phobius) were excluded. We analyzed the predicted secretome of each isolate for candidate effector genes using EffectorP [50].

### Pangenome analyses

We constructed the pangenome based on genes identified in the complete genomes of isolates 1A5, 1E4, 3D1, 3D7, and IPO323. We compared each of the five encoded proteomes with BlastP with a cut-off e-value of 1 × 10^–5^ [82]. We then clustered proteins into families based on pairwise alignments using the software SiLiX v1.2.9 [83] and cut-offs set to 75% identity and 60% coverage. To extrapolate the core genome and pangenome sizes, we performed a resampling of all possible combinations of 1–5 genomes. We modelled the core genome and pangenome size curves by fitting the power law regression formulas: y = Ae^Bx^ + C and y = Ax^B^ + C, respectively, in matlab.

Chromosomal synteny between genomes was analyzed using pairwise blastn on repeat masked genomic sequences. Blastn hits were filtered for a minimum identity of 95%, e-values reported as 0 and a minimum alignment length of 2000 bp. Syntenic regions shared between pairs of homologous chromosomes were visualized using the R package genoPlotR [84].

The chromosomal locations of core, accessory and singleton genes were analyzed in the IPO323 genome for the proximity to transposable elements [53]. Chromosomal locations were also analyzed for euchromatin and heterochromatin marks based on previously generated ChIP-seq datasets for the isolate IPO323 [85]. We accessed the datasets from the public gbrowse server (http://ascobase.cgrb.oregonstate.edu/cgi-bin/gb2/gbrowse/ztritici_public/). For each ChIP-seq sample, we determined significantly enriched domains using the RSEG toolset [86].

### Isolate collection and Illumina genome sequencing

We analyzed Illumina whole-genome sequencing data for 123 *Z. tritici* isolates originating from four worldwide locations (Australia, Israel, Switzerland, and Oregon, USA; Additional file 11: Table S7). Isolates were stored for long-term use in silica at –80 °C. No clonal genotypes were found among these isolates in previous genetic diversity analyses [87]. A total of 106 isolates were previously sequenced on a Illumina HiSeq 2500 (paired-end, 100 bp read lengths) and raw sequencing data is available on the NCBI Short Read Archive under the BioProject PRJNA178194 and PRJNA327615 [42, 44, 88]. We generated paired-end sequencing data for 19 additional isolates following identical procedures as for the previously available data. We deposited the raw sequencing data on the NCBI Short Read Archive under the BioProject PRJNA327615 (see Additional file 11: Table S7 for more details).

### Alignment of Illumina genome sequencing data to complete genomes

We trimmed the raw sequencing reads for quality and adapter contamination using the software Trimmomatic v 0.36 [71], with the following parameters: ILLUMINACLIP:TruSeq3-PE.fa:2:30:10 LEADING:10 TRAILING:10 SLIDINGWINDOW:5:10 MINLEN:50. Trimmed reads were aligned separately on each of the five complete genomes with bowtie2 v2.2.29 [89], with the following parameters: sensitive-local --local –phredd33 –X 1000. We identified and marked PCR duplicates with MarkDuplicates of Picard tools v2.6.0 (http://broadinstitute.github.io/picard).

### Variant calling and analyses

We called SNPs and short indels of all 123 worldwide isolates separately against each of the five complete genome assemblies using the Genome Analysis Toolkit (GATK) v 3.7 pipeline [90]. First, we called variants individually for each isolate using the GATK HaplotypeCaller with the following parameters: -nct 4 --emitRefConfidence GVCF --variant_index_type LINEAR --variant_index_parameter 128000 --sample_ploidy 1. The resulting gvcf files were then combined per reference genome and genotyped with the modules CombineGVCFs and GenotypeGVCFs, respectively. The identified SNPs and short indels were filtered for quality using VariationFiltration with the following cut-offs: QD > 20, FS < 0.1, QUAL > 250, MQ > 30, –2 < BaseQRankSum < 2, –2 < ReadPosRankSum < 2, and –2 < MQRankSumPos < 2. We analyzed the predicted impact of each variant using SnpEff v 4.3 g (with –ud 300) [91]. Variant effects were parsed with SnpSift v4.3 g [92]. To calculate the non-synonymous nucleotide diversity per gene, we extracted non-synonymous SNPs with SnpSift and then used vcftools with the command --haploid --site-pi in vcftools v0.1.14 [93], with the haploid mode patch provided by Julien Y. Dutheil.

### Identification of copy number variation

To detect gene deletions and duplications in the 123 worldwide isolates, we used the software CNVnator [94]. CNVnator uses normalized read depth (RD) to identify copy number variation (CNV). Following standard recommendations, we performed the analyses in bins of 100 bp. Raw CNV calls were filtered using the following criteria: for duplications RD > 2 and deletions RD < 0.4, additional filters were q0 < 0.5, length > 500 bp, and e-value < 0.05. We retrieved genes affected by CNVs using bedtools intersect [95]. CNVnator analyses were performed separately for reads mapped to each of the five complete genomes.

### GO enrichment analysis

Enrichment of GO terms of proteins encoded by genes in different categories was tested using hypergeometric tests with a false discovery rate cut-off set to 0.05. We retained only GO term enrichment results if at least five genes in the genome matched the GO term. All analyses were performed using the R packages GSEABase and GOStats [96].

### Genome assembly and annotation data availability

All complete genome assemblies and corresponding genome annotations were uploaded to the European Nucleotide Archive under accession numbers PRJEB15648, PRJEB20900, and PRJEB20899 for isolates 1A5, 1E4, and 3D1, respectively.

## Additional files

Additional file 1: Table S1. Chromosomal length variation between the five completely assembled genomes of *Zymoseptoria tritici*. (PDF 58 kb)

Additional file 2: Table S2. Assessment of genome and annotation completeness using BUSCO. (PDF 51 kb)

Additional file 3: Table S3. Evaluation of protein clustering thresholds in the construction of the pangenome of *Zymoseptoria tritici*. (PDF 64 kb)

Additional file 4: Table S4. Protein clusterization and functional annotation of the pangenome of *Zymoseptoria tritici*. (XLSX 5334 kb)

Additional file 5: Table S5. Paralogs identified in the pangenome of *Zymoseptoria tritici*. (XLSX 68 kb)

Additional file 6: Table S6. Analyses of genes identified in the isolate IPO323 and classified as accessory or singleton genes in the pangenome. (PDF 67 kb)

Additional file 7: Figure S1. Sequence coverage of Illumina short reads generated from the isolates 3D1, 3D7, 1A5, and 1E4 mapped against the IPO323 genome. For each IPO323 gene, the mean coverage was calculated. The median coverage of all genes of a specific isolate is shown by a vertical bar. (PDF 118 kb)

Additional file 8: Figure S2. Gene ontology enrichment for cellular compartment terms performed for singleton, accessory, and core genes of the *Zymoseptoria tritici* pangenome. (PDF 164 kb)

Additional file 9: Figure S3. Gene ontology enrichment for biological process terms performed for singleton, accessory, and core genes of the *Zymoseptoria tritici* pangenome. (PDF 241 kb)

Additional file 10: Figure S4. Gene ontology enrichment for molecular function terms performed for singleton, accessory, and core genes of the *Zymoseptoria tritici* pangenome. (PDF 235 kb)

Additional file 11: Table S7. Whole-genome sequence analyses of the 123 *Zymoseptoria tritici* isolates included in this study. (XLS 121 kb)

Additional file 12: Table S8. Non-synonymous nucleotide diversity and loss-of-function mutations among 123 *Zymoseptoria tritici* isolates. (XLSX 6650 kb)
