# Supplementary material for: Pangenome analyses of the wheat pathogen Zymoseptoria tritici reveal the structural basis of a highly plastic eukaryotic genome
Source: BMC Biol. 2018 Jan 11;16:5. doi: 10.1186/s12915-017-0457-4 (PMC5765654; doi:10.1186/s12915-017-0457-4)
Supplement: Supplementary file 1 — Chromosomal length variation between the five completely assembled genomes of Zymoseptoria tritici. (PDF 58 kb) [file 12915_2017_457_MOESM1_ESM.pdf]

Table S1 : Chromosomal length variation among the five completely assembled genomes of *Zymoseptoria tritici*. The isolate 1E4 is missing the accessory chromosome 17 and the isolate 3D7 is missing the accessory chromosomes 14, 15, 18 and 21.

Chromosome	Length (kb)					% length variation
	IPO323	1A5	1E4	3D1	3D7	
1	6088.8	6088.5	6061.5	6345.1	6435.9	2.8
2	3860.1	3698.7	3718.6	3863.2	3819.1	2.1
3	3505.4	3797.1	3715.7	3655.3	3904.1	4.0
4	2880.0	2948.4	2972.7	3066.4	2933.1	2.3
5	2861.8	2700.1	2763.3	3035.5	2781.6	4.6
6	2675.0	2700.9	2505.7	2506.1	2415.2	4.8
7	2665.3	2582.1	2654.6	2793.4	2824.7	3.8
8	2443.6	2263.1	2208.4	2353.0	2447.9	4.6
9	2142.5	2048.9	2161.7	2230.2	2020.4	4.0
10	1682.6	1753.5	1847.5	1841.8	1846.0	4.1
11	1624.3	1720.0	1548.2	1659.1	1663.2	3.8
12	1462.6	1468.5	1471.3	1546.8	1367.7	4.4
13	1185.8	1197.7	1157.6	1073.5	1127.2	4.3
14	773.1	677.9	550.9	424.5	-	25.0
15	639.5	653.7	658.4	804.9	-	11.3
16	607.0	611.9	555.8	563.0	624.8	5.2
17	584.1	582.6	-	597.3	578.8	1.4
18	573.7	644.6	649.1	690.7	-	7.6
19	549.8	637.6	599.0	571.2	596.8	5.6
20	472.1	548.9	462.0	546.0	474.9	8.6
21	409.2	374.2	366.6	490.4	-	13.8
Total	39 686.3	39 698.7	38 628.4	40 657.5	37 861.4	2.8
