# Supplementary material for: Pangenome analyses of the wheat pathogen Zymoseptoria tritici reveal the structural basis of a highly plastic eukaryotic genome
Source: BMC Biol. 2018 Jan 11;16:5. doi: 10.1186/s12915-017-0457-4 (PMC5765654; doi:10.1186/s12915-017-0457-4)
Supplement: Supplementary file 2 — Assessment of genome and annotation completeness using BUSCO. (PDF 51 kb) [file 12915_2017_457_MOESM2_ESM.pdf]

Table S2: Assessement of genome and annotation completeness using BUSCO. The total number of Ascomycota BUSCO groups searched was 1315.

Isolate	IPO323	1A5	1E4	3D1	3D7
**Complete**	1287	1292	1290	1294	1273
**Complete (%)**	97.9	98.3	98.1	98.5	96.8
**Complete and single-copy**	1286	1291	1289	1292	1272
**Complete and duplicated**	1	1	1	2	1
**Fragmented**	11	13	12	12	27
**Missing**	17	10	13	9	15
