# Supplementary material for: Pangenome analyses of the wheat pathogen Zymoseptoria tritici reveal the structural basis of a highly plastic eukaryotic genome
Source: BMC Biol. 2018 Jan 11;16:5. doi: 10.1186/s12915-017-0457-4 (PMC5765654; doi:10.1186/s12915-017-0457-4)
Supplement: Supplementary file 3 — Evaluation of protein clustering thresholds in the construction of the pangenome of Zymoseptoria tritici. (PDF 64 kb) [file 12915_2017_457_MOESM3_ESM.pdf]

Table S3: Evaluation of protein clustering thresholds in the construction of the pangenome. The outcomes of the protein clustering runs are reported for different identity and length overlap thresholds. The retained thresholds are marked (*). The pangenome sizes are reported as the total numbers of core (shared by all five genomes), accessory (shared by 2-4 genomes) and singleton genes (found in only one genome).

**Protein clustering cutoffs**	**Identity (%)**	**90**	**80**	**80**	**80**	**75**	**75***	**75**	**60**	**60**	**60**
	**Length overlap (%)**	**90**	**90**	**80**	**70**	**70**	**60***	**50**	**50**	**40**	**30**
**Pangenome composition**	Core genes	7707	7970	8599	8559	8993	9149	9149	9490	9490	9490
	Accessory genes	4832	4628	3928	3968	3498	3377	3377	2825	2825	2825
	Singletons	6256	5430	4406	4406	3831	3223	3223	2581	2581	2581
**Accessory gene numbers**	Accessory genes 1A5	3441	3327	2834	2874	2530	2479	2479	2234	2234	2234
	Accessory genes 1E4	3416	3303	2781	2821	2469	2401	2401	2148	2148	2148
	Accessory genes 3D1	3438	3336	2833	2873	2515	2446	2446	2195	2195	2195
	Accessory genes 3D7	2230	2172	1907	1947	1683	1729	1729	1531	1531	1531
	Accessory genes IPO323	2391	2289	1951	1991	1714	1684	1684	1568	1568	1568
**Singleton gene numbers**	Singleton 1A5	944	795	659	659	569	464	464	368	368	368
	Singleton 1E4	910	760	653	653	571	483	483	395	395	395
	Singleton 3D1	861	700	574	574	498	411	411	321	321	321
	Singleton 3D7	1800	1595	1231	1231	1061	859	859	716	716	716
	Singleton IPO323	1741	1580	1289	1289	1132	1006	1006	781	781	781
	**Total clusters**	18795	18028	16933	16933	16322	15749	15749	14896	14896	14896
