# Supplementary material for: Pangenome analyses of the wheat pathogen Zymoseptoria tritici reveal the structural basis of a highly plastic eukaryotic genome
Source: BMC Biol. 2018 Jan 11;16:5. doi: 10.1186/s12915-017-0457-4 (PMC5765654; doi:10.1186/s12915-017-0457-4)
Supplement: Supplementary file 6 — Analyses of genes identified in the isolate IPO323 and classified as accessory or singleton genes in the pangenome. (PDF 67 kb) [file 12915_2017_457_MOESM6_ESM.pdf]

Table S6: Analyses of genes identified in IPO323 and classified as accessory or singleton genes in the pangenome. The analyses were performed for each of the four other isolates separately. Loss-of-function mutations included early stop codons, loss of exons and deleterious frameshift mutations. Gene deletions were assessed based on short reads mapped against the IPO323 genome. If ≥50% of the gene lacked read coverage, the gene was identified as deleted. A gene was categorized as not expressed based on the normalized CPM (counts per million) over the time course of a wheat infection.

Isolate	1A5	1E4	3D1	3D7
**IPO323 genes not present**	1522	1582	1594	2109
**Genes with loss-of-function mutations**	777	822	748	972
**Gene deletion**	478	545	494	737
**Gene without expression**	1252	1273	1320	1690
**Total genes all categories**	1365	1403	1434	1828
**Total genes all categories (%)**	89.7	88.7	90.0	86.7
