# Supplementary figures and images for: Pangenome analyses of the wheat pathogen Zymoseptoria tritici reveal the structural basis of a highly plastic eukaryotic genome
Source: BMC Biol. 2018 Jan 11;16:5. doi: 10.1186/s12915-017-0457-4 (PMC5765654; doi:10.1186/s12915-017-0457-4)

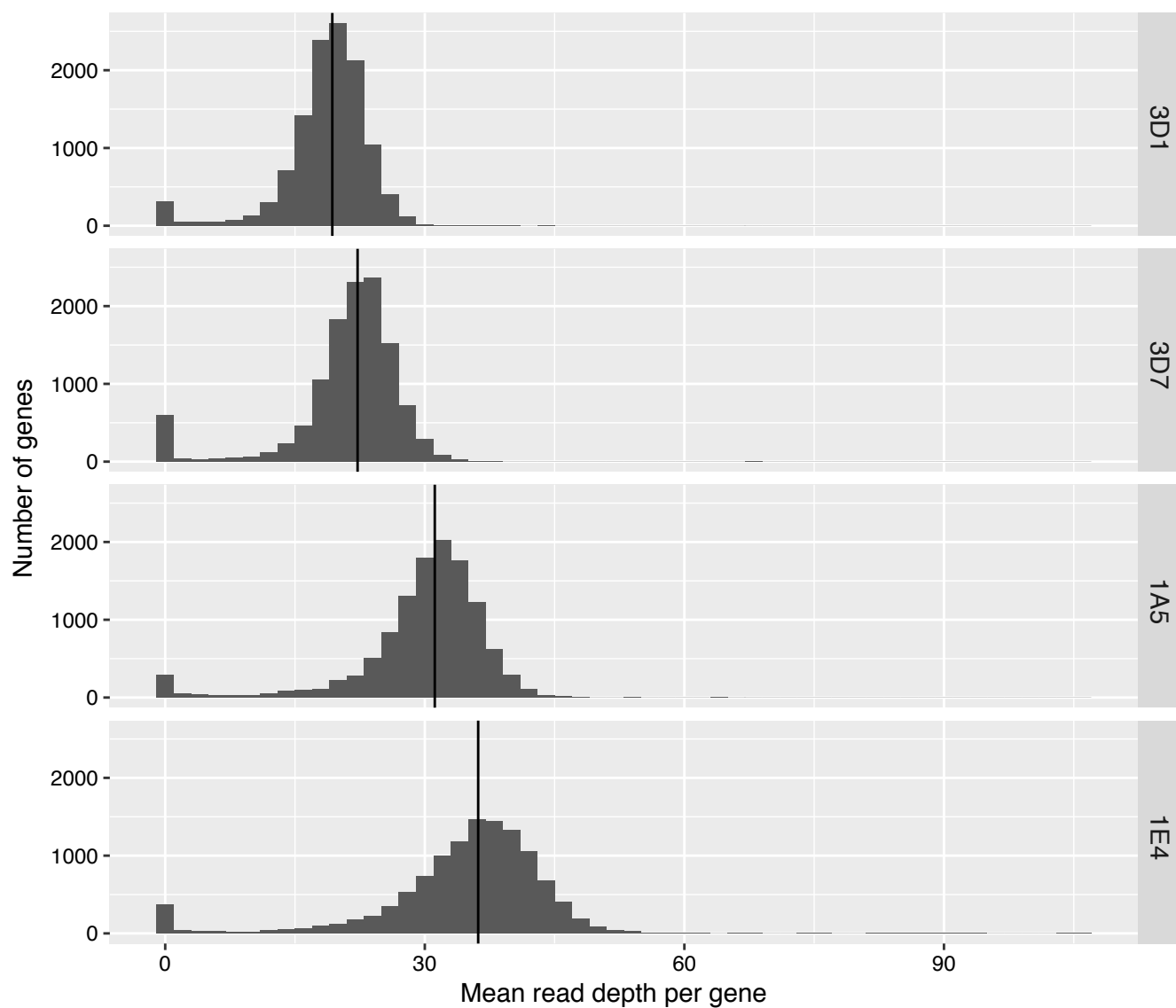

Supplement: Supplementary file 7 — Sequence coverage of Illumina short reads generated from the isolates 3D1, 3D7, 1A5, and 1E4 mapped against the IPO323 genome. For each IPO323 gene, the mean coverage was calculated. The median coverage of all genes of a specific isolate is shown by a vertical bar. (PDF 118 kb) [file 12915_2017_457_MOESM7_ESM.pdf]

Singleton genes

-log10(p-value)

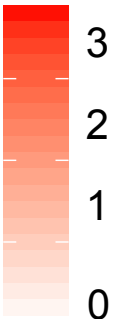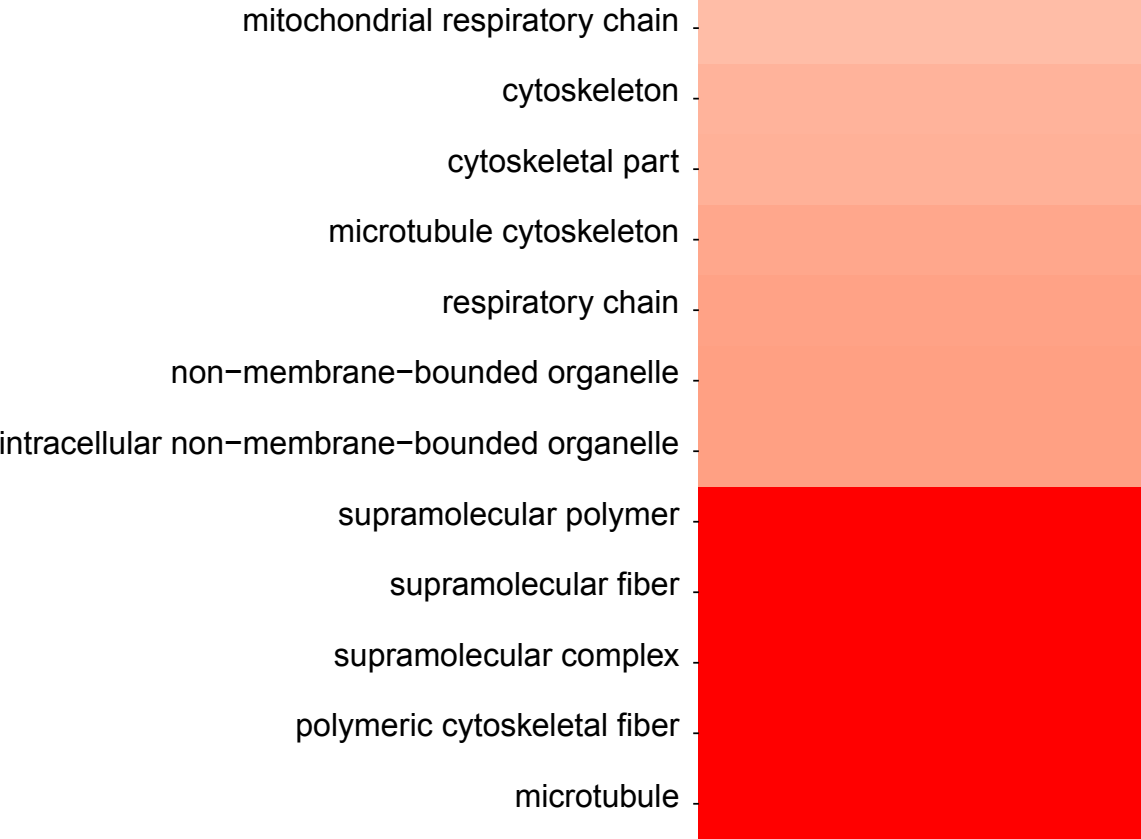

Core genes

-log10(p-value)

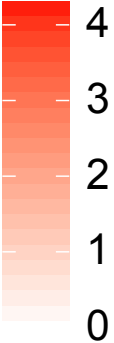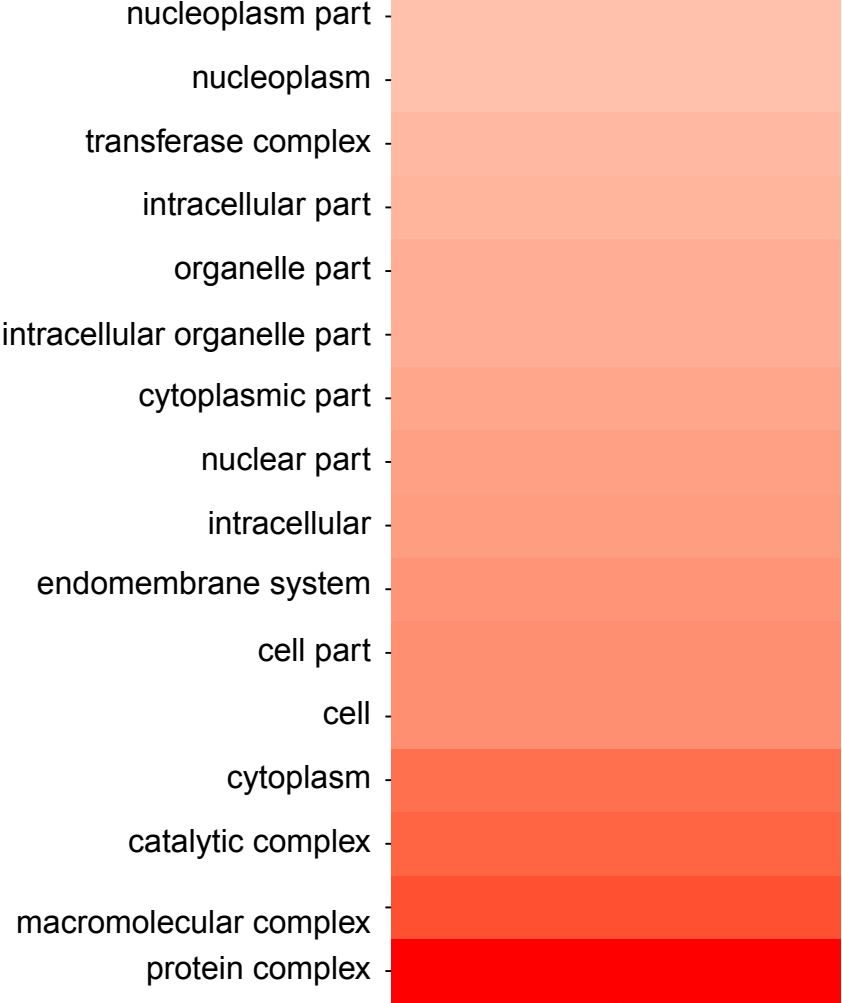

Accessory genes

-log10(p-value)

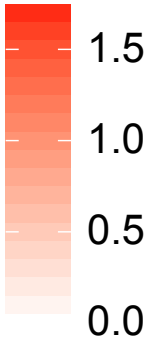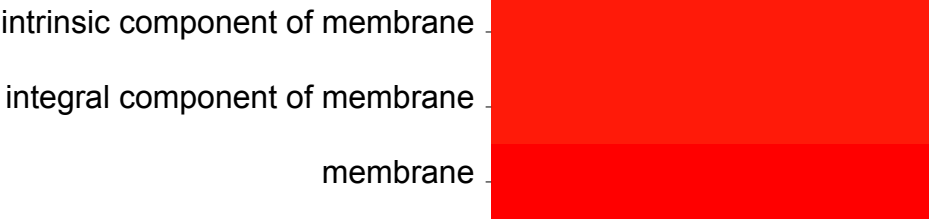

Supplement: Supplementary file 8 — Gene ontology enrichment for cellular compartment terms performed for singleton, accessory, and core genes of the Zymoseptoria tritici pangenome. (PDF 164 kb) [file 12915_2017_457_MOESM8_ESM.pdf]

Singleton genes

-log10(p-value)

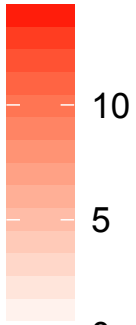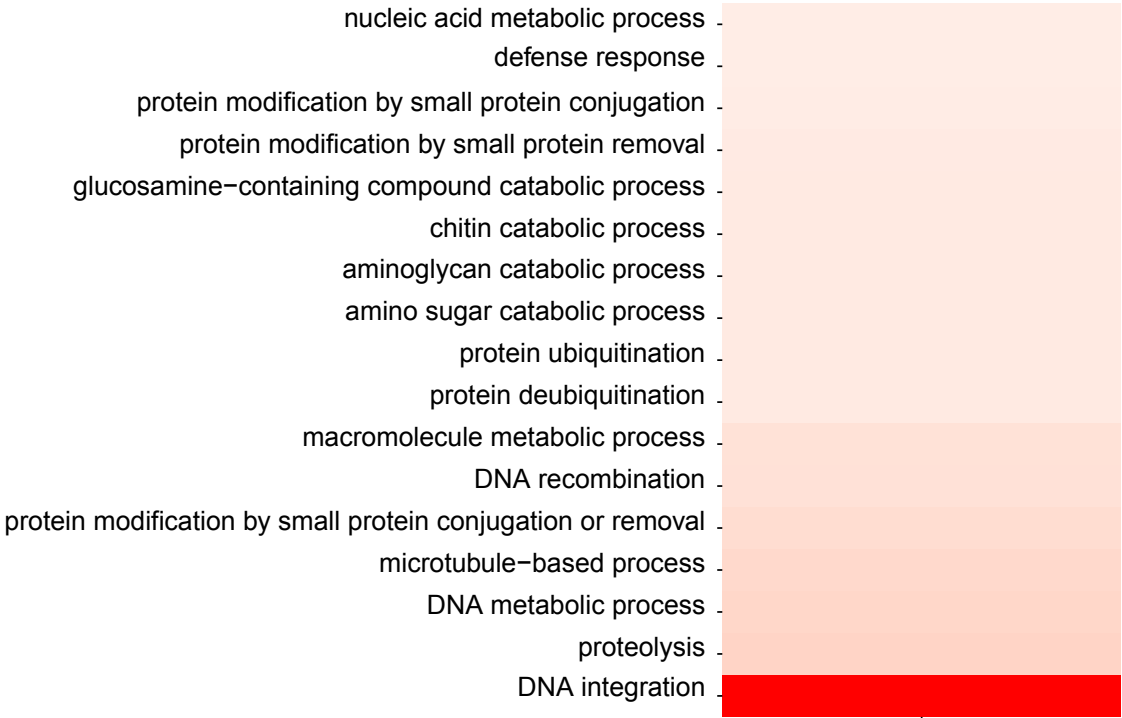

-log10(p-value)

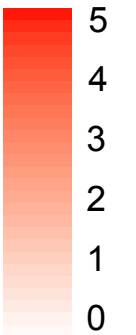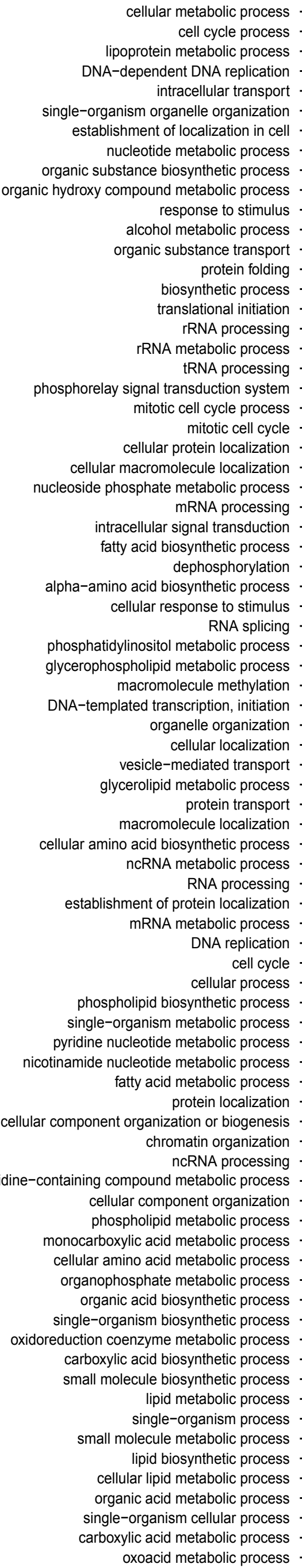

Core genes

Accessory genes

-log10(p-value)

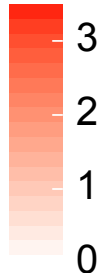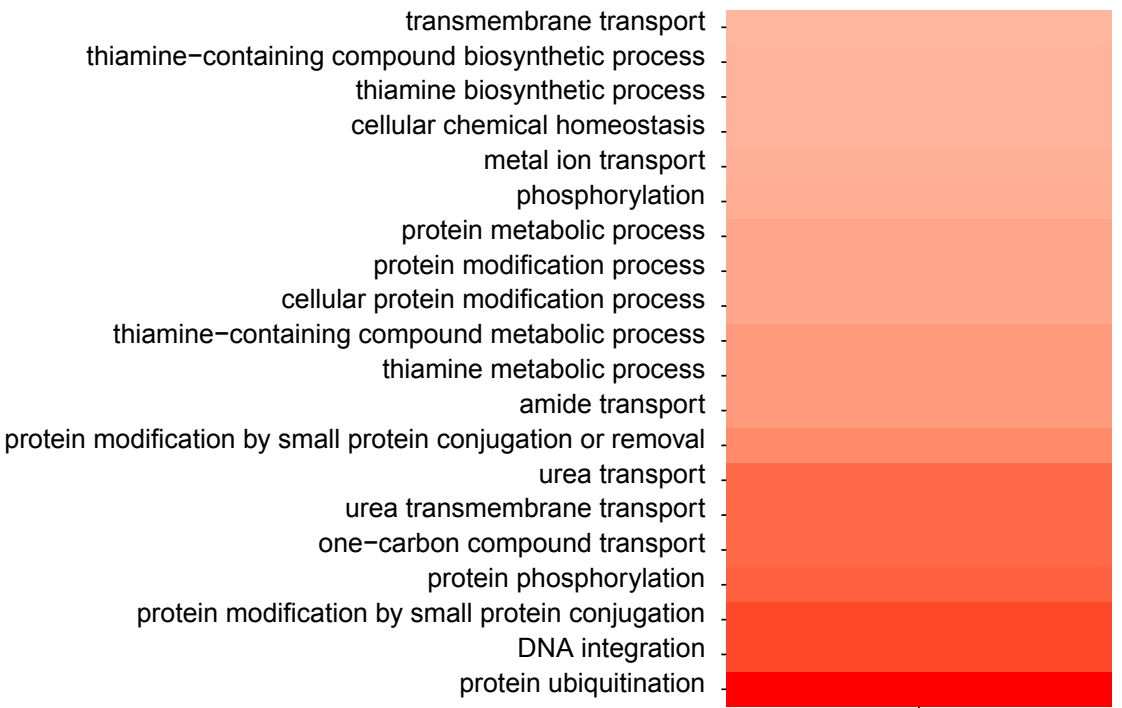

Supplement: Supplementary file 9 — Gene ontology enrichment for biological process terms performed for singleton, accessory, and core genes of the Zymoseptoria tritici pangenome. (PDF 241 kb) [file 12915_2017_457_MOESM9_ESM.pdf]
