# Supplementary material for: Pangenome analyses of the wheat pathogen Zymoseptoria tritici reveal the structural basis of a highly plastic eukaryotic genome
Source: BMC Biol. 2018 Jan 11;16:5. doi: 10.1186/s12915-017-0457-4 (PMC5765654; doi:10.1186/s12915-017-0457-4)
Supplement: Supplementary file 10 — Gene ontology enrichment for molecular function terms performed for singleton, accessory, and core genes of the Zymoseptoria tritici pangenome. (PDF 235 kb) [file 12915_2017_457_MOESM10_ESM.pdf]

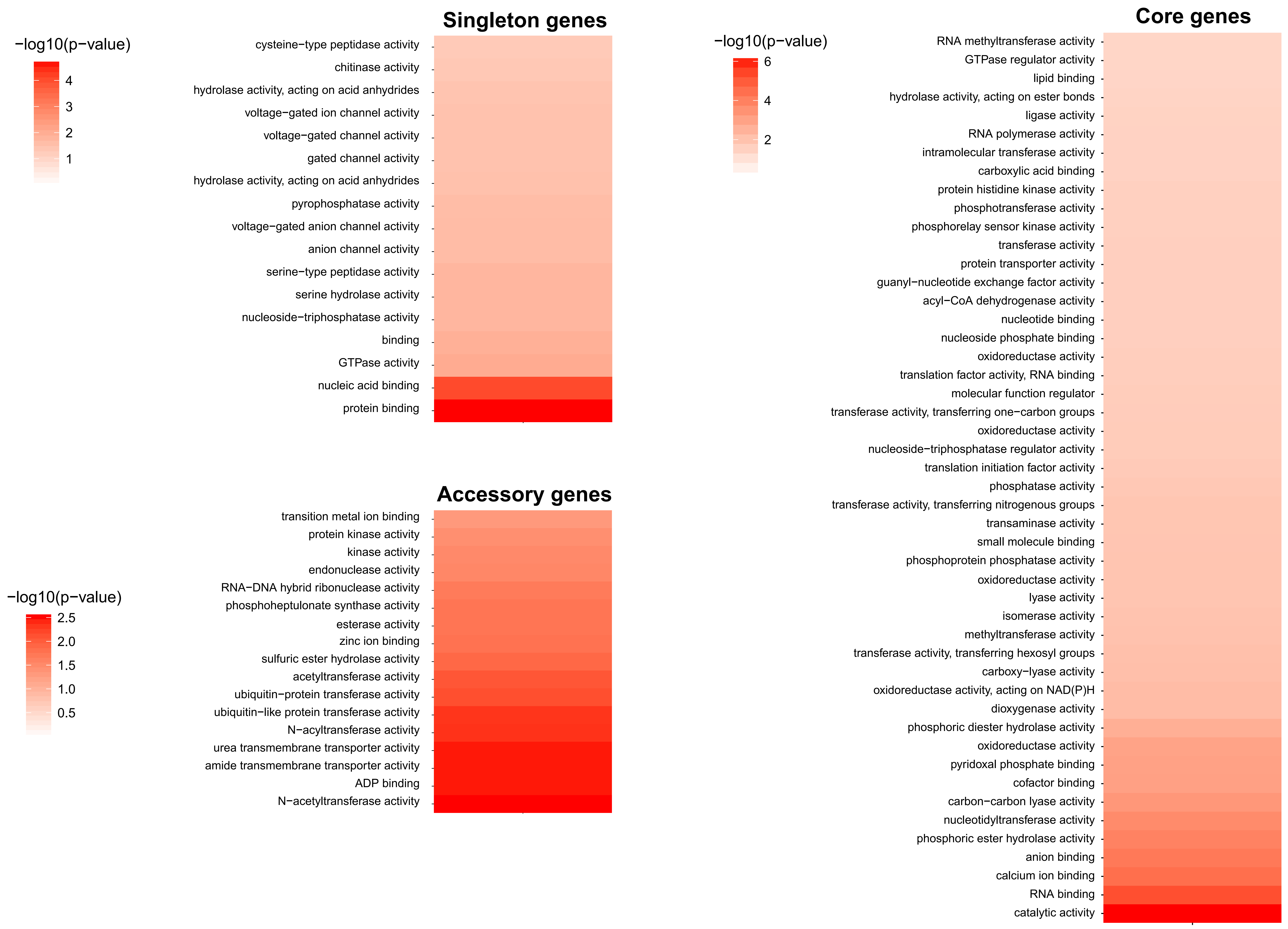
Singleton genes
−log10(p−value)
4
3
2
1
cysteine−type peptidase activity
chitinase activity
hydrolase activity, acting on acid anhydrides
voltage−gated ion channel activity
voltage−gated channel activity
gated channel activity
hydrolase activity, acting on acid anhydrides
pyrophosphatase activity
voltage−gated anion channel activity
anion channel activity
serine−type peptidase activity
serine hydrolase activity
nucleoside−triphosphatase activity
binding
GTPase activity
nucleic acid binding
protein binding
Accessory genes
−log10(p−value)
2.5
2.0
1.5
1.0
0.5
transition metal ion binding
protein kinase activity
kinase activity
endonuclease activity
RNA−DNA hybrid ribonuclease activity
phosphoheptulonate synthase activity
esterase activity
zinc ion binding
sulfuric ester hydrolase activity
acetyltransferase activity
ubiquitin−protein transferase activity
ubiquitin−like protein transferase activity
N−acyltransferase activity
urea transmembrane transporter activity
amide transmembrane transporter activity
ADP binding
N−acetyltransferase activity
Core genes
−log10(p−value)
6
4
2
RNA methyltransferase activity
GTPase regulator activity
lipid binding
hydrolase activity, acting on ester bonds
ligase activity
RNA polymerase activity
intramolecular transferase activity
carboxylic acid binding
protein histidine kinase activity
phosphotransferase activity
phosphorelay sensor kinase activity
transferase activity
protein transporter activity
guanyl−nucleotide exchange factor activity
acyl−CoA dehydrogenase activity
nucleotide binding
nucleoside phosphate binding
oxidoreductase activity
translation factor activity, RNA binding
molecular function regulator
transferase activity, transferring one−carbon groups
oxidoreductase activity
nucleoside−triphosphatase regulator activity
translation initiation factor activity
phosphatase activity
transferase activity, transferring nitrogenous groups
transaminase activity
small molecule binding
phosphoprotein phosphatase activity
oxidoreductase activity
lyase activity
isomerase activity
methyltransferase activity
transferase activity, transferring hexosyl groups
carboxy−lyase activity
oxidoreductase activity, acting on NAD(P)H
dioxygenase activity
phosphoric diester hydrolase activity
oxidoreductase activity
pyridoxal phosphate binding
cofactor binding
carbon−carbon lyase activity
nucleotidyltransferase activity
phosphoric ester hydrolase activity
anion binding
calcium ion binding
RNA binding
catalytic activity
